# Light entrainment of the murine intraocular pressure circadian rhythm utilizes non-local mechanisms

**DOI:** 10.1371/journal.pone.0184790

**Published:** 2017-09-21

**Authors:** Shunsuke Tsuchiya, Ethan D. Buhr, Tomomi Higashide, Kazuhisa Sugiyama, Russell N. Van Gelder

**Affiliations:** 1 Department of Ophthalmology, University of Washington School of Medicine, Seattle, Washington, United States of America; 2 Department of Ophthalmology, Kanazawa University Graduate School of Medical Sciences, Kanazawa, Japan; 3 Department of Pathology, University of Washington School of Medicine, Seattle, Washington, United States of America; 4 Department of Biological Structure, University of Washington School of Medicine, Seattle, Washington, United States of America; Morehouse School of Medicine, UNITED STATES

## Abstract

**Purpose:**

Intraocular pressure (IOP) is known to have a strong circadian rhythm, yet how light/dark cycles entrain this rhythm is unknown. The purpose of this study was to assess whether, like the retina, the mammalian ciliary body and IOP clocks have an intrinsic ability to entrain to light/dark cycles.

**Methods:**

Iris-ciliary body complexes were obtained from *period2*:*luciferase* (PER2::LUC) mice and cultured to measure bioluminescence rhythmicity. Pairs of the iris-ciliary body complex were exposed to antiphasic 9:15 h light/dark cycle *in vitro*. After 4 days of exposure to light/dark cycles, bioluminescence was recorded to establish their circadian phases. In addition, pairs of the iris-ciliary body complex co-cultured with the retinas or corneas of wild-type mice were also investigated. The IOP circadian changes of free-running *Opn4*^*-/-*^;*rd1/rd1* mice whose behavior was antiphasic to wild-type were measured by a rebound tonometry, and compared with wild-type mice. *Opn3*, *Opn4*, and *Opn5* mRNA expression in the iris-ciliary body were analyzed using RT-PCR.

**Results:**

The iris/ciliary body complex expressed *Opn3*, *Opn4*, and *Opn5* mRNA; however, unlike in retina and cornea, neither the iris-CB complex nor the co-cultured complex was directly entrained by light-dark cycle *in vitro*. The diurnal IOP change of *Opn4*^*-/-*^;*rd1/rd1* mice showed an antiphasic pattern to wild-type mice and their rhythms followed the whole-animal behavioral rhythm.

**Conclusions:**

Despite expressing mRNA for several non-visual opsins, circadian rhythms of the iris-ciliary body complex of mice do not entrain directly to light-dark cycles *ex vivo*. Unlike retina, the iris/ciliary body clocks of blind mice remain synchronized to the organismal behavioral rhythm rather than local light-dark cycles. These results suggest that IOP rhythm entrainment is mediated by a systemic rather than local signal in mice.

## Introduction

Intraocular pressure (IOP) in humans and mice has long been known to have a strong circadian rhythm [1–3]. The rhythmicity of IOP is normally entrained to the 24 h light/dark cycle in mouse [3], rat [4], rabbit [5], and human [6]. Interestingly, IOP is higher in the dark phase irrespective of diurnal or nocturnal habits of animals. Elevated IOP is a strong risk factor for primary open-angle glaucoma, which is one of the leading causes of the blindness worldwide. Abnormal fluctuation of the IOP through 24 h period may contribute to a progression of glaucoma [7]; thus understanding the diurnal regulation of the IOP rhythm may provide mechanistic insights into glaucoma and its management.

The molecular and cellular mechanisms of the IOP rhythm are not well understood. Maeda et al. showed that mice lacking the core clock genes *Cry1* and *Cry2* lost their IOP rhythm [8], suggesting this rhythm depends on the core clock mechanism [9]. The diurnal rhythm of intraocular pressure is thought to derive at least in part from changes in ciliary body aqueous production in both animals [10] and humans [11,12]. Similarly, Dalvin et al. showed that the iris-ciliary body complex of mice have robust rhythmic core clock gene expression, which strongly correlated with IOP diurnal curve [13].

In mammals, most tissues have autonomous circadian clocks, and these circadian oscillations are coordinated by the suprachiasmatic nuclei (SCN) in the hypothalamus to generate integrated rhythms of physiology and metabolism [14]. The circadian systems of the mammals function as a hierarchy in which the central pacemaker, the SCN, orchestrates the peripheral tissues located throughout the body via hormonal and neural signaling [15]. The SCN are entrained by external photic input through photoreception in the retina [16] and their signaling is mediated by melanopsin (OPN4)-expressing retinal ganglion cells (intrinsically photosensitive retinal ganglion cell; ipRGC). Mice that lack of ipRGCs show free running circadian rhythms of behavior under light-dark cycles, with free-running periods typically shorter than 24-hours [17]. While behavioral rhythms of mice lacking both melanopsin and rods/cones (*Opn4-/-;rd1/rd1* mice) similarly do not entrain to light:dark cycles, surprisingly, the retinal circadian rhythms of these animals remain entrained to light [18]. This local circadian entrainment is mediated by the rhabdomeric opsin, neuropsin (OPN5), which appears both necessary and sufficient for local retinal circadian entrainment [19]. Another atypical opsin, encephalopsin (OPN3) is also expressed in retina, while not required for light entrainment of the retina, does contribute to the amplitude of circadian rhythms in that tissue [16].

While, surprisingly, cornea also expresses OPN5 and has a directly light-entrainable circadian clock [16], it is unclear at present whether other ocular tissues express OPN3 and/or OPN5 and whether local circadian entrainment by light occurs in these tissues. Given the known rhythmicity of ciliary body function and IOP, we performed this study to determine whether the local clock of the mouse ciliary body and the rhythm of IOP are locally entrained by light-dark cycles.

## Methods

### Animals

C57Bl/6J (WT; Jackson laboratory, Bar Harbor, ME, USA) and *Opn4-/-;rd1/rd1* mice were used in this study. They were 3–6 months old and males and females were randomly selected. The *Opn4-/-;rd1/rd1* mice were generated by crossing the *rd1/rd1* line [20] (C57Bl/6 background from the Jackson Laboratory) and *Opn4*^*-/-*^ line [21] (backcrossed to C57Bl/6J background for more than seven generations). To monitor the bioluminescence, all mice except for WT mice that were used for IOP measurements were bred into the PER2::LUC background [22]. Mice that were utilized for IOP measurements were kept in 12:12 light/dark cycle (see below). Otherwise, mice were kept in 14:10 light/dark cycle and light was provided by fluorescent tubes (~120 μW/cm^2^ at cage level). All animals were treated in accordance with the ARVO Statement for the Use of Animals in Ophthalmic and Vision Research and all animal experiments were conducted following approved protocols by the institutional animal care and use committee at the University of Washington.

### Quantitative RT-PCR

WT mice were euthanized by CO_2_ aspiration. Retinas, corneas, and iris-CB complexes were immediately dissected from the eyes in cold HBSS (Life technologies) and were placed in RNAlater (Qiagen). Livers were dissected directly into RNAlater. RNA was purified from tissues by using TRI Reagent (Sigma-Aldrich) following the manufacturer’s protocol. 200 ng total RNA from each sample was used to generate cDNA using High-Capacity cDNA Reverse Transcription Kit (Applied Biosystems).

Quantitative RT-PCR was performed by using Absolute Blue SYBR Green Low Rox master mix (Thermo Fisher) and an Applied Biosystems 7500 Real Time PCR system. The obtained cDNA was amplified with specific primer pairs described below. The PCR products were assessed by melt curve and gel electrophoresis using 2.0% agarose gels to confirm appropriate amplification of the targets. Each gene expressions were determined by standard curve method. To compare gene expression of *Opn3* and *Opn5* among tissues, their expressions were normalized by beta-actin mRNA expression in the same sample and then compared to those levels to a liver sample. The following PCR primers were used: Opn4 (5’-TCT GTT AGC CCC ACG ACA TC-3’ and 5’-TGA ACA TGT TTG CTG GTG TCC-3’), Opn3 (5’-CTG TTC GGA GTC ACC TTC AC-3’ and 5’-GTA TGT CTA GGA TGT ACC TGT TC-3’), Opn5 (5’-AGC TTT TGG AAG GCC AGA C-3’ and 5’-CAG CAC AGC AGA AGA CTT C-3’), and beta-actin (5’-AGG TGA CAG CAT TGC TCC TG-3’ and 5’-GCT GCC TCA ACA CCT CAA C-3’). The conditions of the PCR were as follows: 95°C for 10 min for the initial denaturation followed by 40 cycles of 15 sec at 95°C for denaturation and 30 sec for annealing/ extension. The annealing temperature was at 60°C for Opn3, Opn4, and beta-actin and at 66°C for Opn5.

### Real-time bioluminescence recording and analysis of circadian rhythm

*PER2*::*LUC* or *Opn4*^*-/-*^;*rd1/rd1*;*PER2*::*LUC* mice were euthanized by CO_2_ aspiration 2–3 h before lights off (i.e. zeitgeber time 9–10) and tissues were immediately dissected in cold HBSS solution (Life technologies). Iris-ciliary body (iris-CB) complex and cornea were cultured on cell culture inserts (PICMORG50; Millipore) in sealed dishes with DMEM media containing B-27 supplement (Life technologies), 352.5 μg/ml sodium bicarbonate, 10mM HEPES (Life technologies), 25 U/ml penicillin, 25 μg/ml streptomycin (Life technologies), and 0.1 mM luciferin potassium salt (Biosynth). Retinas were cultured on cell culture inserts in Neurobasal medium (Life technologies) containing B-27 supplement (Life technologies), 25 U/ml penicillin, 25 μg/ml streptomycin (Life technologies) and 2 mM L-Gln (Life technologies) in 5% CO_2_ up to 24 h. The retinas were then transferred to sealed dishes with the same DMEM media described above. For co-culture experiments, cornea and retina were dissected from WT mice in the same procedure as described above, and then they were placed on the cell culture inserts with the iris-CB complex dissected from PER2::LUC mice. All tissue cultures were incubated at 37°C, and their bioluminescence was recorded for 1 min at 7.5 min intervals using a Lumicycle luminometer (Actimetrics). The obtained bioluminescence was detrended by using a polynomial fit line to eliminate a steady decline of background bioluminescence. Period of oscillation was decided by best-fit sine wave analysis in the Lumicycle Analysis software.

### *Ex vivo* light/dark cycles

For exposing pairs of tissues from the same animal to light/dark cycle *ex vivo*, the tissues were placed on the opposite sides (0° and 180°) of a clock apparatus as previously described [18]. Briefly, a rotating shutter created antiphasic 9:15 light-dark cycles for each tissue. In addition of the DMEM media described above, all cultures for this ex vivo light/dark entrainment experiments were maintained in the presence of 10 μM 9-*cis*-retinaldehyde. Light was provided by using an array of five light-emitting diode (LED) sets within the incubator with peak wavelength at 417 nm, 475nm, and 530nm. White light was generated by turning on the 417-nm, 475-nm, and 530-nm LEDs simultaneously. All radiometric measurements were assessed with a Macam Q203 quantum radiometer (Macam Photometrics), and spectral irradiance was measured with a SpectroCal spectral radiometer (Cambridge Research Systems). After 4 days of light/dark cycle *ex vivo*, the tissues were placed in the luminometer and their luminescence was recorded as described above. The first peak in the sine wave fit analysis was used for a phase marker of each tissue.

### Intraocular pressure measurements

IOP was measured with a rebound tonometer (Icare TonoLab; Colonial Medical supply, Franconia, NH, USA) as previously described [23]. Unanesthetized mice were gently held using a decapiCone (Braintree Scientific, Inc., Braintree, MA, USA) and a custom made restrainer. IOP diurnal curve was obtained by measuring IOP every 4 h at ZT 2, 6, 10, 14, 18, and 22 (lights on at ZT 0 and lights off at ZT 12). IOP measurements during the light phase were conducted under room lighting condition and the measurements during the dark phase were performed under dim red light condition. At each time point, eight IOP measurements (four measurements in each eye) were recorded and the average of the values was reported as IOP of the animal at that time point and used for further statistical analysis.

To determine whether the IOP rhythm of the mice is primarily controlled by light or an SCN-controlled internal timing cue, we utilized *Opn4*^*-/-*^;*rd1/rd1* mice which do not entrain to 12:12 light/dark cycle and show free-running behavioral rhythm in monitoring locomotor activities [15, 18]. *Opn4*^*-/-*^;*rd1/rd1* mice were kept in individual cages with running wheels under 12:12 light/dark conditions. Light was supplied by the same LED system utilized in the ex vivo entrainment experiment (~4 W/m^2^). Locomotor activities and light/dark cycles were recorded by Clock Lab data collection system (Actimetrics). As the free-running period of these mice is less than 24 hours, their activity onset varies with respect to the phase of the light dark cycle and will eventually become antiphasic with wild-type animals (i.e., the *Opn4*^*-/-*^;*rd1/rd1* mice become active at ZT 0). On that day, IOP of the *Opn4*^*-/-*^;*rd1/rd1* mice were measured repeatedly. The same procedures were also performed on WT mice that were entrained for more than two weeks in 12:12 light/dark cycle.

### Statistical analysis

To compare the pattern of diurnal IOP rhythm of WT and *Opn4*^*-/-*^;*rd1/rd1*, two-way repeated measures analysis of variance (ANOVA) was performed. IOP differences at each time points were analyzed by Student’s t-test with Bonferroni corrections for multiple comparisons. All statistical analyses were performed with SPSS version 23.0 (SPSS, Chicago, IL, USA). P<0.05 was considered to indicate statistical significance.

## Results

### Gene expression of *Opn3*, *Opn4*, and *Opn5* in iris-ciliary body complex

**Fig 1:**
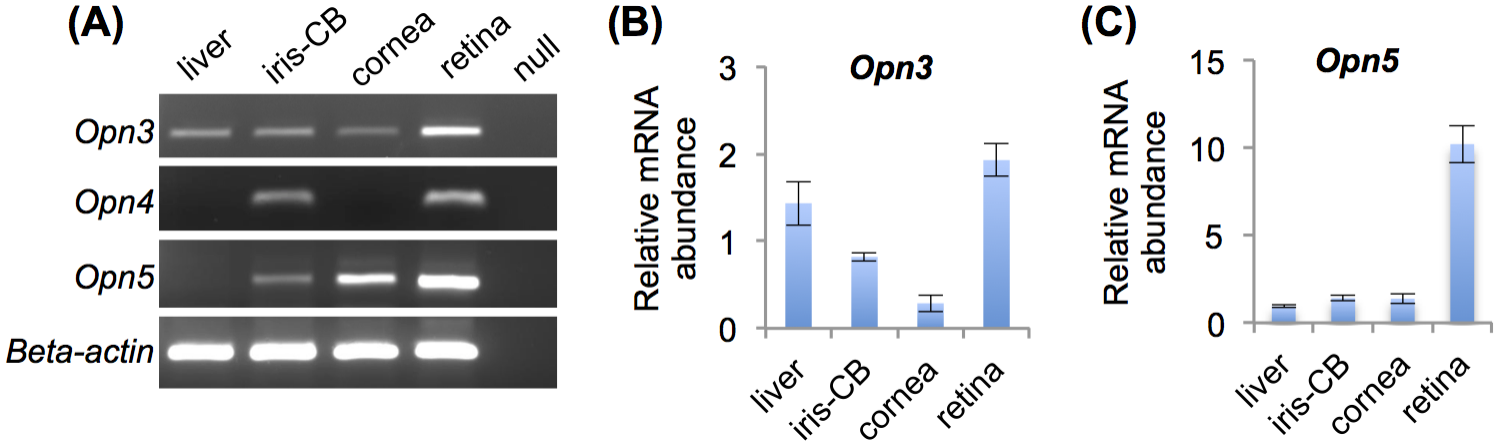
The gene expression of *Opn3*, *Opn4*, and *Opn5* in the iris-ciliary body complex. (A) Detection of *Opn3*, *Opn4*, and *Opn5* in liver, iris-CB, cornea, and retina. Relative abundance of *Opn3* (B) and *Opn5* (C) mRNA was compared with beta-actin levels and compared with liver expression based on quantitative RT-PCR results. Error bars represent 1 SEM (n = 3 for each).

RT-PCR detected *Opn3*, *Opn4*, and *Opn5* mRNA in WT mouse iris-CB complex (Fig 1A, 1B and 1C). Quantitation through qPCR showed that *Opn5* mRNA was expressed at a level comparable to that of cornea (although substantially less than expressed in retina), while *Opn3* mRNA appears more strongly expressed in iris-CB than in cornea, although again less than in retina. The relative abundance of the melanopsin mRNA was not shown because we could not detect any signals from mouse liver or cornea in the real-time PCR in this study.

### Circadian rhythms of PER2::LUC bioluminescence in cultured iris-ciliary body

**Fig 2:**
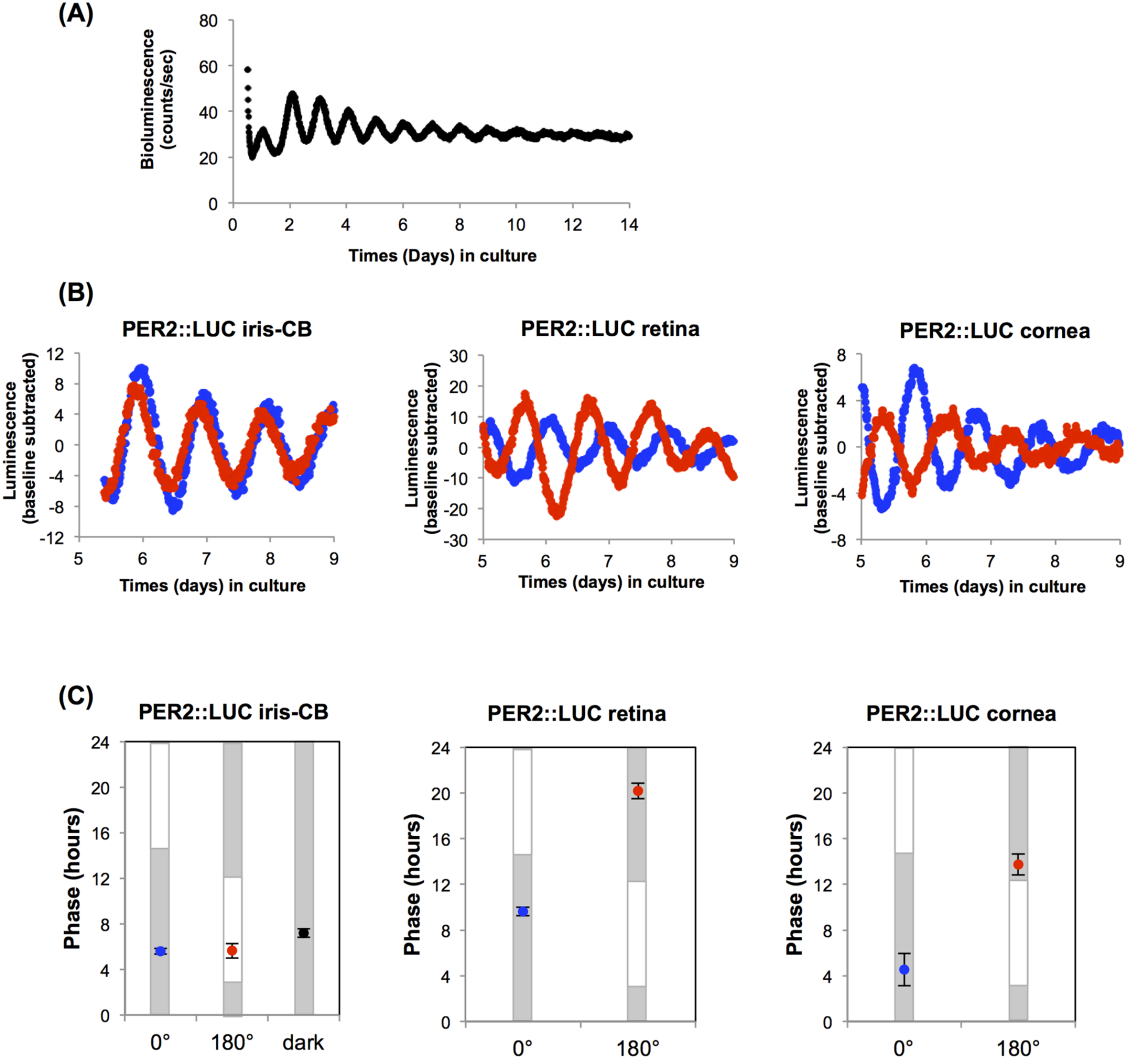
The iris-ciliary body (CB) does not entrain to light/dark cycle *in vitro*. (A) Circadian PER2::LUC rhythm in the cultured iris-CB was recorded with a luminometer for 14 days. (B) Representative records of bioluminescence from PER2::LUC iris-CB (left), retina (middle), and cornea (right) in a light/dark exposure experiment. PER2::LUC luminescence traces with background luminescence subtracted by using a polynomial fit line. Red and blue dots represent two separate cultures of the tissues from the same PER2::LUC mouse. Traces were recorded after 4 days light/dark exposure ex vivo in 0° (blue) and 180° (red) position of in a clock apparatus. (C) Average time of the peak PER2::LUC luminescence on the first day of the recording in a luminometer after 4 days exposure of light/dark cycle in vitro. Blue and red indicate the 0° and 180° position in the clock apparatus, respectively. Error bars represent 1 SEM. Gray bars indicate periods of the day when tissues were previously kept in the dark and white bars indicate times of 6 W/m^2^ white lights. Black point represents the average phase of untreated iris-CB complex in the fifth day in culture. PER2::LUC iris-CB: control (dark), n = 4; 0°, n = 4; 180°, n = 4, retina: 0°, n = 4; 180°, n = 4, cornea: 0°, n = 4; 180°, n = 4, respectively.

A representative measurement of the circadian Per2::LUC activity of an iris-CB complex is illustrated in Fig 2A. Robust circadian rhythms of iris-CB complex Per2^luciferase^ bioluminescence were observed from all explants. The average circadian period *in vitro* was 24.2 ± 0.4 hours (mean ± standard deviation, n = 5).

**Fig 3:**
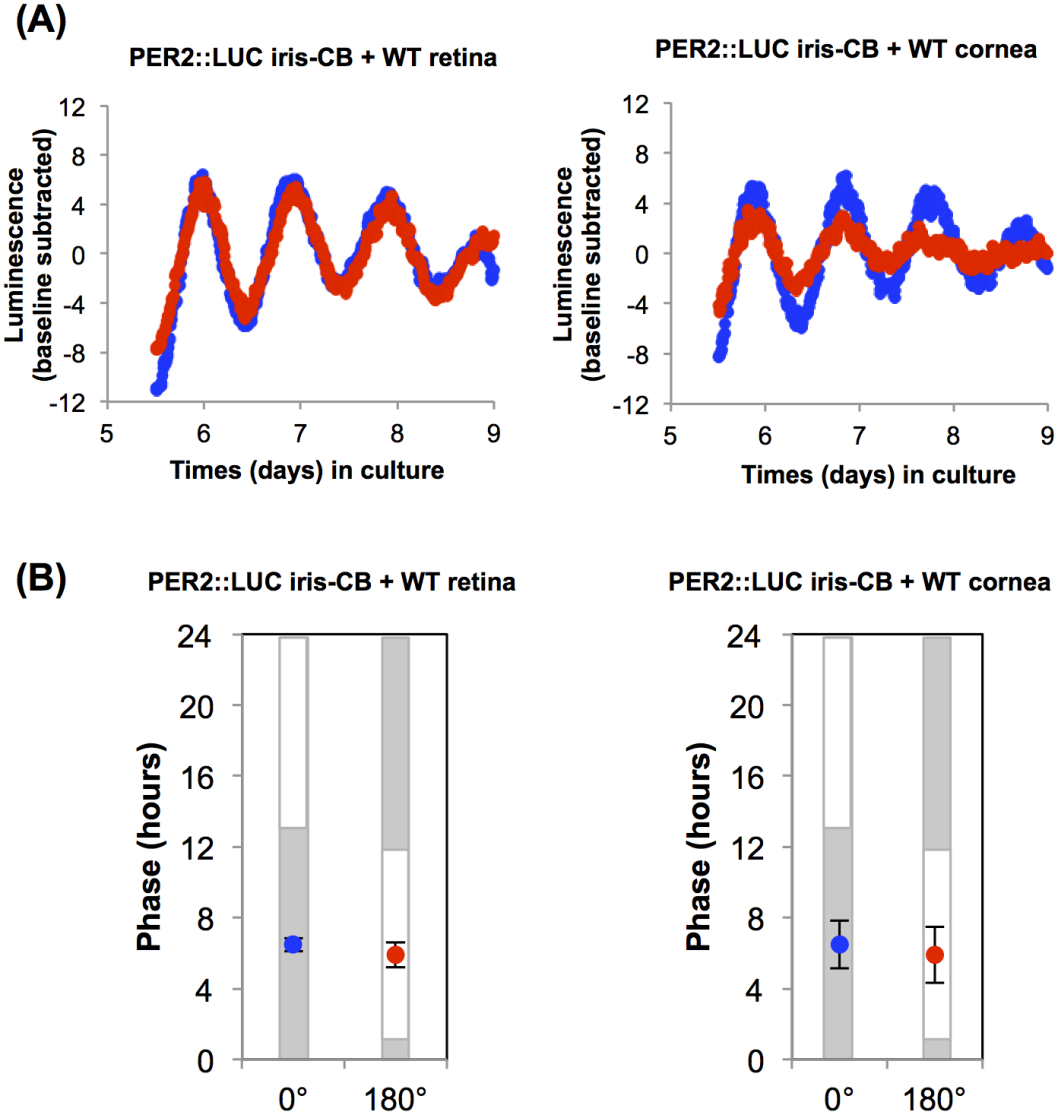
The iris-ciliary body (CB) co-cultured with retina or cornea is not entrained by light/dark cycle in vitro. (A) Representative records of bioluminescence from PER2::LUC iris-CB co-cultured with WT retina (left) and cornea (right). PER2::LUC luminescence traces with background luminescence subtracted by using a polynomial fit line. Red and blue dots represent two separate cultures of the tissues from the same PER2::LUC mice. Traces were recorded after 4 days light/dark exposure ex vivo in 0° (blue) and 180° (red) position of in a clock apparatus. (B) Average time of the peak PER2::LUC luminescence on the first day of the recording in a luminometer after 4 days exposure of light/dark cycle in vitro. Blue and red indicate the 0° and 180° position in the clock apparatus, respectively. Error bars represent 1 SEM. Gray bars indicate periods of the day when tissues were previously kept in the dark and white bars indicate times of 4 W/m^2^ light exposure from 417 nm LED. PER2::LUC iris-CB + retina, n = 3; PER2::LUC iris-CB + WT cornea, n = 3, respectively.

As iris-CB showed *Opn3* and *Opn5* mRNA expression, we performed *ex vivo* light entrainment experiments to determine whether the iris-CB is capable of local photic entrainment like retina or cornea [18,19]. Retinal and corneal explants demonstrated clear entrainment to light dark cycles (Fig 2B and 2C), while the phases of the iris-ciliary body complex exposed to the anti-phasic light/dark cycle were not synchronized to the *ex vivo* light/dark cycle. We also conducted *ex vivo* light entrainment experiment of the iris-ciliary body complex co-cultured with retina and cornea, to determine if local paracrine signals from these tissues could entrain the iris-CB. However, none of the co-cultured iris-ciliary body complexes showed photic entrainment after four days exposure to the light/dark cycle when co-cultured with either wild-type retina or cornea (Fig 3A and 3B).

### Intraocular pressure rhythm of WT and *Opn4*^*-/-*^;*rd1/rd1* mouse

**Fig 4:**
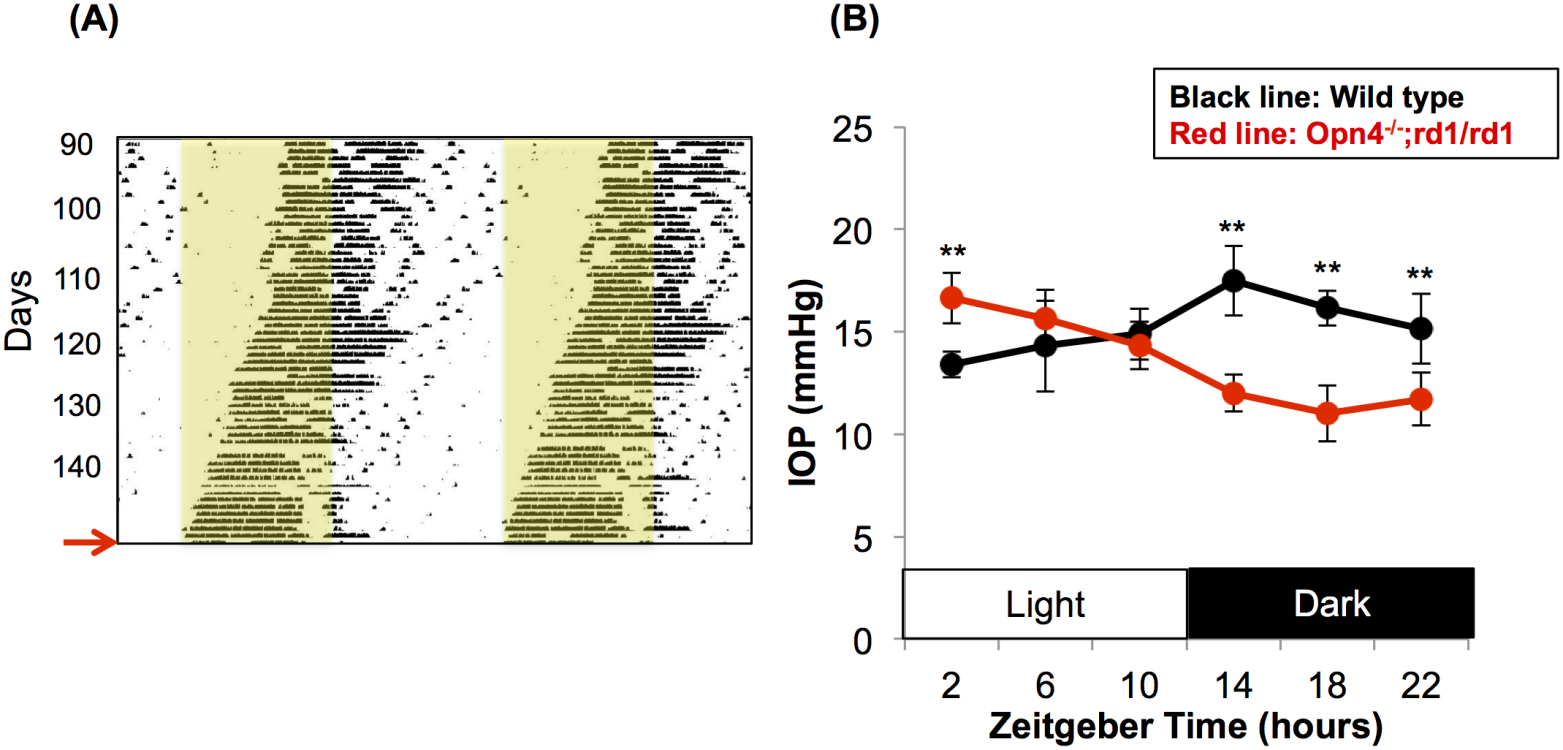
Intraocular pressure (IOP) clocks were synchronized with the behavioral rhythm. On the day at which the *Opn4*^*-/-*^;*rd1/rd1* mice became active at ZT 0 (i.e. antiphasic to the WT mice), their IOP was measured repeatedly. The IOP of the WT mice that were kept in the 12 h Light/dark cycle was also measured at the same time points. (A) A representative actogram of wheel-running behavior of an *Opn4*^*-/-*^;*rd1/rd1* mouse. Black indicates time of day when the mouse was running in the wheel, and yellow area indicates times of day when lights were on. On the day at which the *Opn4*^*-/-*^;*rd1/rd1* mice became active at the time of light-on (indicated by a red arrow), IOP measures were begun. (B) Mean IOP curves showed significant fluctuation in both groups (P = 0.009, two-way repeated measures ANOVA). Black line, wild type (n = 8); red line, *Opn4*^*-/-*^;*rd1/rd1* (n = 5). **P<0.01; student’s t-test with Bonferroni corrections. Error bars indicate standard deviation.

Due to lack of rods, cones, and melanopsin, *Opn4*^*-/-*^;*rd1/rd1* mice show free-running behavioral circadian rhythms with a period of approximately 23.6 hours under light-dark conditions [15]. Thus, the free-running period of behavior will advance by about 0.5 hour daily. 24-h IOP patterns of *Opn4*^*-/-*^;*rd1/rd1* mice were obtained on the day when they were antiphasically active relative to the WT mice (i.e. active in light phase, Fig 4A). Under these conditions, retinal circadian rhythms will remain synchronized to the light-dark cycle, while SCN rhythms will free-run in phase with behavior [18]. Both WT and *Opn4*^*-/-*^;*rd1/rd1* mice showed a robust circadian IOP rhythm under 12h light/dark cycle (Fig 4B, P = 0.009). However, the patterns of IOP curves were significantly different from each other (P<0.001). IOP of the WT was lowest at ZT 2 (13.4 ± 1.2 mmHg), the early in the light phase, and was highest at ZT 14 (17.5 ± 0.9 mmHg; P<0.001; n = 8), early in the dark period, respectively. Conversely, the IOP curve of the *Opn4*^*-/-*^;*rd1/rd1* mice tested when behavioral rhythms demonstrated activity onset at lights-on mice showed trough at ZT 18 (10.9 ± 0.9 mmHg), the middle of the dark phase, and the peak at ZT 2 (16.7 ± 0.7 mmHg; P = 0.016; n = 5), the early in the light phase. The level of IOP was significantly different between the two groups at ZT2, ZT14, ZT18, and ZT22 (P <0.001, <0.001, <0.001, and = 0.012, respectively).

## Discussion

In the present study, we demonstrate that the local circadian clock of the ciliary body does not entrain to light/dark cycle *ex vivo*, either in isolation or when co-cultured with retina or cornea. Moreover, when the circadian phase of behavior was desynchronized from the light-dark cycle (in *Opn4*^*-/-*^;*rd1/rd1* mice), the IOP rhythm remained synchronized to behavior and not local light signals. These findings suggest that, despite expressing three non-visual opsin mRNAs, the iris-ciliary body responds to primarily to exogenous entraining signals, likely from the central circadian pacemaker, and not to local light-driven signals.

We found that the mouse iris-CB complex expressed *Opn5* mRNA in the present study. In birds, OPN5 (neuropsin) acts as a deep brain photoreceptor in the hypothalamus of birds and has been thought to be involved in seasonal reproduction [24,25]. Buhr et al. recently reported that cornea and retina may be entrained directly by light *in vitro* without cones, rods, and melanopsin, but that OPN5 is critical to this local photic entrainment in mammals [18,19]. In our study, we saw weak expression of *Opn5* mRNA in the iris-CB complex compared to retina and cornea. It is possible that iris-CB does not have sufficient neuropsin expression to mediate photic input locally within the tissue. Alternatively, it could be expressed in a small subset of cells which might be directly light sensitive, but are not sufficient to synchronize other PER2-expressing cells in the entire tissue. We also found *Opn3* and *Opn4* mRNA expression in WT mouse iris-CB. Xue T et al. have suggested that local photoreception by melanopsin in the iris may directly mediate a component of the pupillary light response [26]. However, our data suggest that melanopsin is not sufficient to mediate local entrainment of the iris-CB circadian clock, as wild-type tissue did not entrain *ex vivo*. The same argument also suggests that potential photopigment OPN3 is similarly not sufficient for local entrainment. The function of OPN3 in mammals is still unknown even though its broad expression among mammalian tissues [27,28]. Presuming that mRNA expression indicates protein expression of these photoreceptive molecules in the iris-CB, their function remains to be determined, but does not appear to include entrainment of the circadian clock.

Our experiments were designed to specifically test the hypothesis that light might directly entrain the circadian clock in the ciliary body of mice, and thereby entrain the circadian rhythm of intraocular pressure. We find that, unlike in retina or cornea (tissues which also express novel opsins such as OPN5), light does not directly entrain iris-CB tissue *in vitro*. Thus, entrainment is non-local. The source of the entraining signal is presently unclear, but our finding that co-culture of retina or cornea with iris-CB does not lead to synchronization suggests that local signals from these tissues are not sufficient to mediate entrainment. It is possible that other ocular sites may provide local entraining signals.

Alternatively, systemic humoral signals may be responsible. The SCN, the mammalian master clock, appears to play a key role in synchronizing the IOP rhythm since SCN lesion disrupts IOP fluctuation [29] and chemical stimulation of the dorsomedial hypothalamus and surrounding perifornical area, which receive projections from the SCN, induces IOP elevation [30]. The sympathetic nervous system is one candidate for mediating signals to generate IOP rhythm because unilateral cervical sympathetic ganglionectomy abolished IOP rhythm of the ipsilateral eye in rabbits [31]. Also, timolol, a non-selective beta-adrenergic receptor antagonist, significantly reduces IOP only when applied the dark phase [32]. Nevertheless, deletions of alpha adrenergic receptors [33] or beta adrenergic receptors [34] did not disturb circadian regulation of IOP; thus adrenergic receptors alone are less likely to be responsible for the circadian regulation of IOP. Melatonin also has been studied for its role in diurnal IOP regulation. In mammals, melatonin is generated in circadian manner and topical application of melatonin or a melatonin analogue significantly reduced IOP at night [35]. However, mice used in the present study (i.e. C57Bl/6 background) are genetically deficient in melatonin production [36], so that melatonin may be dispensable for the entrainment of IOP. Another possible candidate is glucocorticoid, which is rhythmically secreted by adrenal glands and contribute to the peripheral clock synchronization in the mammalian body [37,38]. The non-pigmented epithelium of the ciliary body and the trabecular meshwork, the ocular structures which participate in the aqueous humor dynamics, have glucocorticoid receptors [39] and topical or systemic application of steroids may result in an IOP elevation [40]. Further research is needed to determine the signaling pathways that mediate systemic cues from SCN to ciliary body.

The current study has a number of limitations. We cannot exclude that the dissection of iris-CB does not injure a light-entrainment mechanism (although the free-running circadian clock mechanism appears to survive dissection, and similar dissection of retina and cornea leaves OPN5-dependent entrainment mechanisms intact). In our experiments utilizing *Opn4-/-;rd1/rd1* mice, we measured the circadian rhythm of IOP as outcome. The relative importance of circadian rhythmicity within the ciliary body to observed IOP rhythms has not been demonstrated (i.e. measuring a ciliary-body specific clock gene knockout animal’s IOP rhythm). It is possible that the ciliary body remained synchronized to the light-dark cycle *in vivo* while other factors affecting IOP (such as trabecular outflow and uveoscleral outflow) were synchronized; were this the case we might have expected the amplitude of the circadian IOP rhythm to be reduced (which was not observed) but we cannot rule out desynchrony of the component flows determining IOP.

In conclusion, the mouse iris-CB expresses mRNA encoding several potential opsins, including *Opn5* mRNA; however, the mouse iris-CB complex does not entrain to local light/dark cycles *ex vivo* and remains synchronized to the behavioral rhythmicity rather than local light-dark cycle in visually blind mice. Taken together these data suggest that, unlike in retina and cornea, iris-CB and associated IOP rhythm are not entrained locally within the eye, but rely on synchronizing signals from the suprachiasmatic nucleus or other sites. Further study is necessary to determine the function of novel opsins in iris and ciliary body, and to identify the systemic signals driving the circadian rhythm of IOP.
